# Permutation entropy is not an age-independent parameter for EEG-based anesthesia monitoring

**DOI:** 10.3389/fnagi.2023.1173304

**Published:** 2023-06-15

**Authors:** Darren Hight, David P. Obert, Stephan Kratzer, Gerhard Schneider, Pablo Sepulveda, Jamie Sleigh, Paul S. García, Matthias Kreuzer

**Affiliations:** ^1^Department of Anaesthesiology and Pain Medicine, Inselspital, Bern University Hospital, University of Bern, Bern, Switzerland; ^2^Department of Anesthesiology and Intensive Care Medicine, School of Medicine, Technical University of Munich, Munich, Germany; ^3^Department of Anesthesia, Critical Care and Pain Medicine, Massachusetts General Hospital, Boston, MA, United States; ^4^Department of Anesthesia, Harvard Medical School, Boston, MA, United States; ^5^Department of Anesthesia and Intensive Care Medicine, Hessing Foundation, Augsburg, Germany; ^6^Department of Anesthesiology, Hospital Base San José, Osorno/Universidad Austral, Valdivia, Chile; ^7^Department of Anaesthesia, Waikato Clinical School, University of Auckland, Hamilton, New Zealand; ^8^Department of Anesthesiology, Columbia University, New York, NY, United States

**Keywords:** age, anesthesia, EEG, hypnosis, permutation entropy

## Abstract

**Background:**

An optimized anesthesia monitoring using electroencephalographic (EEG) information in the elderly could help to reduce the incidence of postoperative complications. Processed EEG information that is available to the anesthesiologist is affected by the age-induced changes of the raw EEG. While most of these methods indicate a “more awake” patient with age, the permutation entropy (PeEn) has been proposed as an age-independent measure. In this article, we show that PeEn is also influenced by age, independent of parameter settings.

**Methods:**

We retrospectively analyzed the EEG of more than 300 patients, recorded during steady state anesthesia without stimulation, and calculated the PeEn for different embedding dimensions m that was applied to the EEG filtered to a wide variety of frequency ranges. We constructed linear models to evaluate the relationship between age and PeEn. To compare our results to published studies, we also performed a stepwise dichotomization and used non-parametric tests and effect sizes for pairwise comparisons.

**Results:**

We found a significant influence of age on PeEn for all settings except for narrow band EEG activity. The analysis of the dichotomized data also revealed significant differences between old and young patients for the PeEn settings used in published studies.

**Conclusion:**

Based on our findings, we could show the influence of age on PeEn. This result was independent of parameter, sample rate, and filter settings. Hence, age should be taken into consideration when using PeEn to monitor patient EEG.

## Introduction

Monitoring the patient under general anesthesia using the electroencephalogram (EEG) has become a standard procedure in many hospitals. All commercially available monitoring systems exploit information from the EEG in the frequency domain. The algorithms are proprietary, so the way the index is generated remains largely unknown. For the bispectral index (BIS, Medtronic, Dublin, Ireland) a reverse engineering approach has identified the higher frequencies in the EEG as a main contributor to the index. What all indices have in common, is their “*one-size-fits-all*” approach for adult patients, in that no patient demographic factors or substance information are considered when the processed EEG index is calculated. This “*one-size-fits-all*” approach is vulnerable to age-related changes in the EEG (Ni et al., 2019; Obert et al., 2021, 2023). The indices of most systems increase with age (Ni et al., 2019; Obert et al., 2021, 2023) as do their included frequency bands or sub-parameters (Schultz et al., 2004; Purdon et al., 2015; Kreuzer et al., 2020). Other, entropic approaches applied to the EEG in the time domain also seem affected by age (Kreuzer et al., 2020).

In contrast to these findings, a more recent publication found no significant impact of age on the permutation entropy (PeEn) and the authors concluded that in contrast to other investigated parameters, PeEn seems to be a promising candidate for an “age-independent” measure (Biggs et al., 2022). In that study, these contradictory findings (Kreuzer et al., 2020) were explained by different settings of the PeEn embedding dimension as well as time point and sampling issues. To shed some light into this issue, we reanalyzed some of our previously collected data used to show the age-dependency of EEG parameters. We show that PeEn is indeed affected by age, and that the reported findings of Biggs et al. (2022), showing no significant age-related effect, can be attributed to small sample sizes and dichotomization of the age groups.

## Materials and methods

We reanalyzed data from two studies used previously for evaluation of age-induced effects of processed EEG parameters (Kreuzer et al., 2020; Obert et al., 2023). Therefore, we decided to use EEG segments that were recorded during a state of unstimulated anesthesia right before incision.

### Reanalyzed EEG data

The data of the first study (study 1) was initially published in the British Journal of Anaesthesia (Hesse et al., 2019). As in our initial analysis investigating the impact of age on PeEn (Kreuzer et al., 2020), we used data from patients that received propofol for anesthesia induction and sevoflurane for anesthesia maintenance. In short, single channel EEG was recorded with either a bispectral index or an entropy module. To align sample rates, we resampled the BIS data to a sampling frequency of 100 Hz. The EEG segment that was used to evaluate the impact of age was 20 s in length (2000 data points) and obtained between the 5th and 2nd minute before incision, i.e., at a state of general anesthesia without surgical stimulation. Details can be found in the initial paper explaining age-induced changes on entropic parameters (Kreuzer et al., 2020).

The data of the second study (study 2) was initially published in the Journal of Clinical Anesthesia (Lutz et al., 2022). Again, the extracted data was of 30 s length (7500 data points) and stems from the 2 – 10 min before immediately incision, when the patients were either maintained with propofol, sevoflurane or desflurane, to analyze the EEG obtained during a general anesthesia without surgical stimulation situation. EEG was recorded with a 10-channel montage and at a sampling rate of 250 Hz. Details can be found in the paper explaining age-induced changes on the processed EEG index of the SEDLine monitor (Obert et al., 2023).

The use of both data sets allows for analyses independent of sample rate, anesthetic agent, or EEG setup.

For both data sets we could show that the age-adjusted MAC did not significantly change with age and that the propofol concentration significantly decreased with age.

### EEG analysis

**FIGURE 1:**
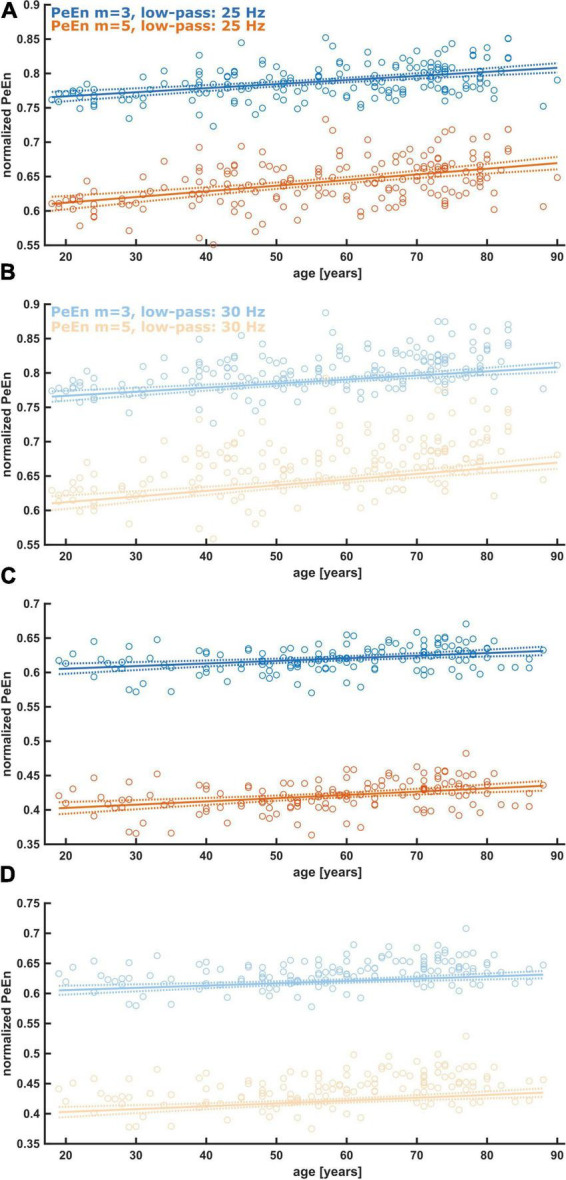
Linear regression models describing the relationship between age and normalized PeEn for the previously used settings, i.e., an embedding dimension of *m* = 3 or *m* = 5, and a low-pass filter set to 25 or 30 Hz. **(A)** Results from patients from study 1. The normalized PeEn significantly increased with age for *m* = 3. **(B)** Results from patients from study 1. The normalized PeEn significantly increased with age for *m* = 5. **(C)** Results from patients from study 2. The normalized PeEn significantly increased with age for *m* = 3. **(D)** Results from patients from study 2. The normalized PeEn significantly increased with age for *m* = 5.

All included EEG episodes were free of artifacts and showed no burst suppression activity. In this analysis we wanted to test the effect of different high- and low-pass filter settings as well as different embedding dimensions of PeEn on the relationship between PeEn and age. The algorithm of PeEn was introduced by Bandt and Pompe (2002) and calculates the Shannon entropy of rank order patterns of length m. In general, PeEn has been proposed to reflect the complexity and the information content of a signal, but some results suggest that this may overestimate the interpretability of PeEn, at least for a *m* = 3 (Berger et al., 2017). The main differences in parameter settings between the Biggs article (Biggs et al., 2022) and our own earlier findings (Kreuzer et al., 2020; Obert et al., 2021) were the sample rate (89 Hz vs. 100 Hz), the embedding dimension (*m* = 5 vs. *m* = 3), the low pass cut-off (25 Hz vs. 30 Hz), and the maintenance anesthetic (propofol vs. sevoflurane). Different time points were also used. Nonetheless, if PeEn is truly an age-independent parameter, it should work at all anesthetic levels in general. In the following analysis we calculated the PeEn using *m* from 3 to 5 and for all frequencies from 1 to 30 Hz with a minimal bandwidth of 1 Hz, using a 4th order forward-backward filter routine (*filtfilt* in Matlab). For the evaluation of dichotomized data, we calculated PeEn for the EEG low pass filtered at 30 or at 25 Hz. We did not change the time delay tau in the PeEn algorithm but kept tau = 1 because higher tau values may lead to aliasing (Berger et al., 2017). For the results presented in Figure 1, we used the normalized PeEn for display purposes. Therefore, we followed the normalization as used by Bandt and Pompe (2002) and divided PeEn by log2(*m*!).

### Statistical analysis

To evaluate an age-induced effect on PeEn, we calculated linear regression models (PeEn = k*age + *y*, with *k* being the slope of the model and *y* the intercept) for all m values and all filter settings using the Matlab function *fitlm*. To evaluate a significant trend of PeEn with age, this function uses a *t*-test to test the hypothesis that the slope of the model equals 0. We have already used this approach in our earlier publications to avoid dichotomizing the data with an arbitrary age threshold choice.

But to compare our findings with studies that use dichotomized data, we also conducted paired tests, by stepwise including younger and older patients, as well as testing a fixed age threshold of 65 years. This means we started with a pairwise comparison of the 10 youngest and 10 oldest patients in the cohort and then added the next youngest or oldest for the next comparison. The last comparison was then the young half versus the old half in the group. We performed Mann–Whitney U tests because of varying sample sizes, and we also calculated the *area under the receiver operating curve* (AUC) with 10k-fold bootstrapped 95% confidence interval using the Matlab-based MES toolbox (Hentschke, 2011). As a rule of the thumb, AUC > 0.7 or AUC < 0.3 indicates a clinically relevant effect (Mandrekar, 2010). The effect size and confidence intervals can also help to prevent drawing wrong conclusions of “no effect” or “no difference” because of a non-significant result (Amrhein et al., 2019). We used the AUC as effect size to evaluate the degree of separation between the old and young patients. Hence, we were interested in the absolute difference of the AUC from the AUC = 0.5, indicative of “no effect.” This is why we also present AUC values below 0.5.

## Results

### Frequency band analysis

As a first step we evaluated the impact of age on the reported settings and could show that for a *m* = 3 and *m* = 5 and low pass filter settings of 25 or 30 Hz, the (normalized) PeEn significantly increased with age as presented in Figure 1 and Table 1.

**TABLE 1:** Parameters for the linear models (PeEn = slope*age + intercept) using the normalized PeEn for the settings of *m* = 3 or *m* = 5 and the low pass set to either 25 or 30 Hz.

		Slope	Intercept	*t*-stat	*p*-value	*R* ^2^
Study 1:	PeEn *m* = 3 (25 Hz)	0.001	0.755	6.60	<0.001	0.20
	PeEn *m* = 3 (30 Hz)	0.001	0.762	7.17	<0.001	0.22
	PeEn *m* = 5 (25 Hz)	0.001	0.596	6.82	<0.001	0.21
	PeEn *m* = 5 (30 Hz)	0.001	0.608	7.24	<0.001	0.23
Study 2:	PeEn *m* = 3 (25 Hz)	0.001	0.755	6.60	<0.001	0.20
	PeEn *m* = 3 (30 Hz)	0.001	0.762	7.17	<0.001	0.22
	PeEn *m* = 5 (25 Hz)	0.001	0.596	6.82	<0.001	0.21
	PeEn *m* = 5 (30 Hz)	0.001	0.608	7.24	<0.001	0.23

**FIGURE 2:**
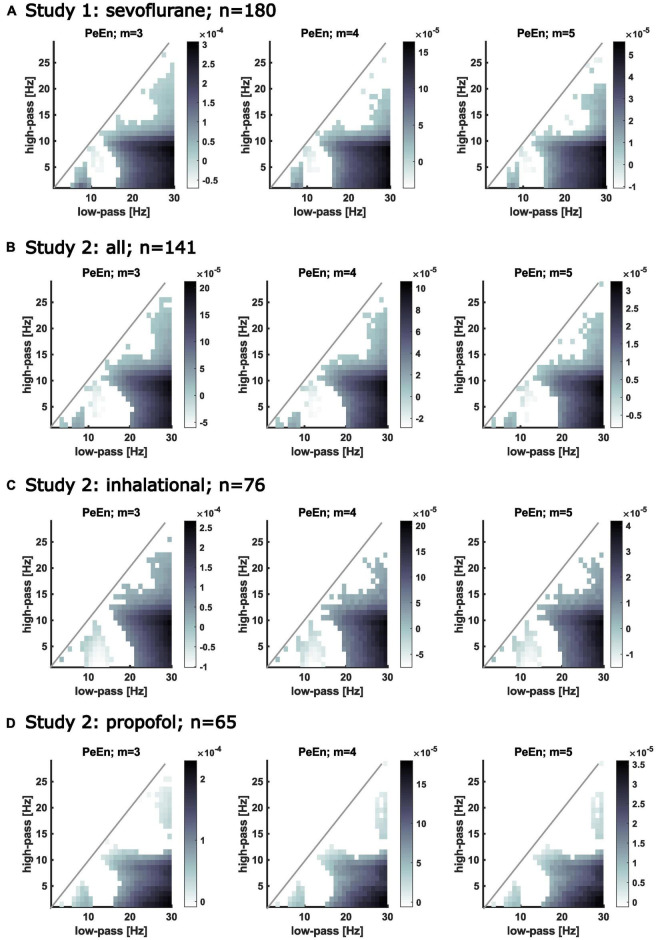
Matrices showing the slopes of the calculated linear regression models describing the relationship between PeEn and age for different frequency bands. The filter settings are indicated on the *x*-axis (low-pass) and on the *y*-axis (high-pass). If the regression model for the PeEn of the EEG filtered to the indicated range showed a significant change with age, the corresponding pixel in the matrix is colored. The darker the color, the steeper is the slope, i.e., the stronger PeEn was affected by age for that filter setting. White pixels below the diagonal indicate no significant change in PeEn with age. The area above the diagonal remains white because of invalid filter settings, i.e., the high-pass cutoff frequency would be higher than the low-pass cutoff frequency. Significant relationships between age and PeEn also shown for different embedding dimensions for the bandpass filtered EEG. Age affected PeEn most strongly for the wide frequency ranges (e.g., high-pass filters below 5 Hz with a low-pass above 20 Hz). (A) Results from patients from study 1. All received propofol induction and maintenance with sevoflurane. (B) Results from all patients from study 2. All received propofol induction and maintenance with sevoflurane, desflurane, or propofol. (C) Results from patients from study 2 that received an inhalational anesthetic for anesthesia maintenance. (D) Results from patients from study 2 that received propofol for anesthesia maintenance.

For a more generalized picture, we calculated the linear regression models for different m and different EEG frequency bands. For the majority of the band pass filter settings we found a significant relationship between age and PeEn irrespective of the embedding dimension m. The steepest slopes of the model, i.e., the strongest age-PeEn associations, could be found for the wide frequency bands, and when beta activity (around 15–30 Hz) was retained in the EEG signal. Non-significant trends between PeEn and age were generally observed when low-frequency low-pass filters were applied or when the band-pass filter was narrow. This means that the typical settings used to investigate age-related changes (Kreuzer et al., 2020; Biggs et al., 2022) and the settings used to investigate the performance of PeEn to monitor anesthesia (Jordan et al., 2008; Olofsen et al., 2008; Schneider et al., 2014) were affected the strongest by age. This is seen as the dark gray regions in Figure 2 (showing the results from study 1 and 2). Table 2 complements this figure with the presentation of the parameter of the linear models.

**TABLE 2:** Parameters of the linear regression model (PeEn = slope*age + intercept).

		Intercept	Slope	*p*	*R* ^2^
Study 1:	PeEn *m* = 3 (25 Hz)	1.9594	0.0002	<0.001	0.17
	PeEn *m* = 4 (25 Hz)	3.0061	0.0005	<0.001	0.18
	PeEn *m* = 5 (25 Hz)	4.1309	0.0008	<0.001	0.19
	PeEn *m* = 3 (30 Hz)	1.9713	0.0003	<0.001	0.21
	PeEn *m* = 4 (30 Hz)	3.0409	0.0006	<0.001	0.22
	PeEn *m* = 5 (30 Hz)	4.1957	0.0009	<0.001	0.22
Study 2:	PeEn *m* = 3 (25 Hz)	1.5338	0.0002	<0.001	0.09
	PeEn *m* = 4 (25 Hz)	2.0887	0.0004	<0.001	0.10
	PeEn *m* = 5 (25 Hz)	2.6715	0.0007	<0.001	0.11
	PeEn *m* = 3 (30 Hz)	1.5524	0.0002	<0.001	0.12
	PeEn *m* = 4 (30 Hz)	2.1334	0.0005	<0.001	0.13
	PeEn *m* = 5 (30 Hz)	2.7469	0.0008	<0.001	0.13

### Dichotomized data

**FIGURE 3:**
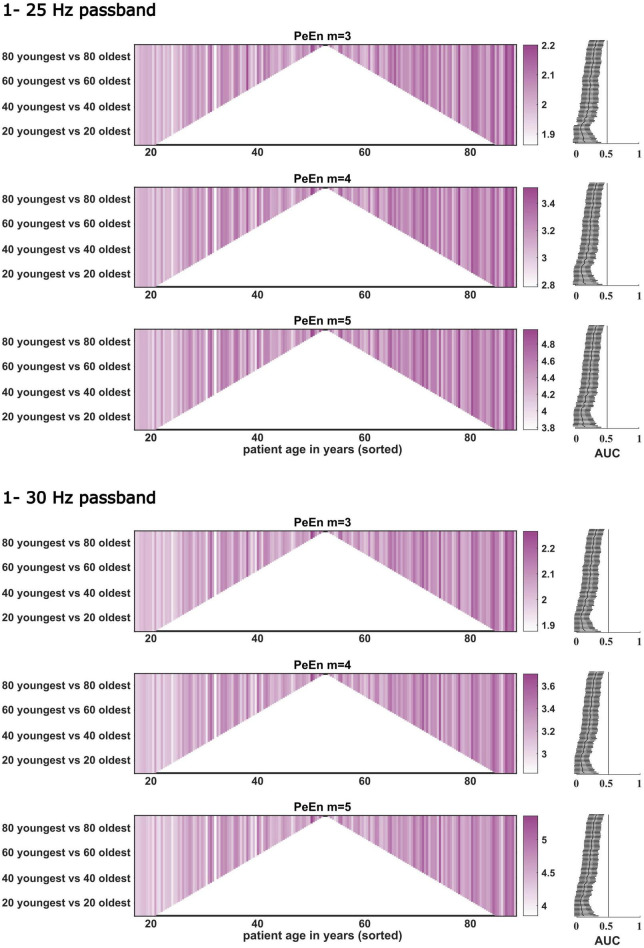
Dichotomous comparisons between the “young” and “old” patients for PeEn with different embedding dimensions for the EEG bandpass filtered to either 1–25 or 1–30 Hz for all patients from study 1 (*n* = 180). Patients are sorted by age on the *x*-axis. Darker colors indicate a higher PeEn. The comparisons started with the youngest 10 vs. the oldest 10 patients (at the bottom of the plot) and then one young and older patient was included in a stepwise manner up to the 89 youngest vs. the 89 oldest, a dichotomization around the median. For all comparisons we found a significant difference in PeEn values between young and old as indicated by the AUC with 95% confidence intervals shown next to the comparisons to the right. The vertical line in these plots indicates AUC = 0.5. If this line is not crossed by the confidence intervals, the difference in PeEn between “young” and “old” can be considered significant.

**FIGURE 4:**
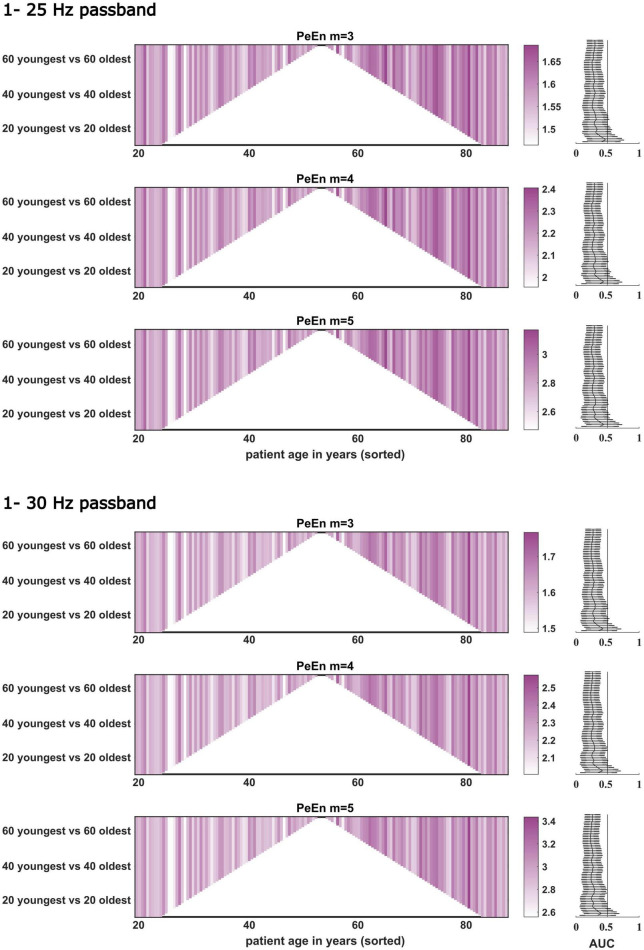
Dichotomous comparisons between the “young” and “old” patients for PeEn with different embedding dimensions for the EEG bandpass filtered to either 1–25 or 1–30 Hz for all patients from study 2 (*n* = 141). The comparisons started with the youngest 10 vs. the oldest 10 patients and then one young and old was included in a stepwise manner up to the 69 youngest vs. the 69 oldest. For most comparisons, we found a significant difference between young and old as indicated by the AUC with 95% confidence intervals. If this line is not crossed by the confidence intervals, the difference in PeEn between “young” and “old” can be considered significant.

For our stepwise approach, where we started with the comparisons of the mean PeEn values in the youngest ten versus the oldest ten patients for each study and finished with the comparison of the “young” half versus the “old” half of the patients, we found significant differences for each comparison as indicated by a *p* < 0.05 with the Mann–Whitney U test and by the AUC confidence intervals not containing 0.5 for study 1 (Figures 3, 4). In these figures, PeEn values are shown for each patient (color of the vertical lines), and can be seen to generally increase (becoming darker) with increasing patient age. The analysis of the data from study 2, confirmed this result. Only at the first steps (with the 25 Hz cutoff: first 9 steps for *m* = 3; first 7 steps for *m* = 4 and *m* = 5; with the 30 Hz cutoff: first 5 steps for all m), when comparing the small samples of the “youngest” versus the “oldest” we found non-significant differences (Figures 3, 4). For both studies the age cutoff for the equal sized groups (i.e., 89 vs. 89 patients in study 1, and 69 vs. 69 patients in study 2) was 59 years for the “young” group and 60 years for the “older” group. Because the threshold of 65 years is often used and suggested for a person to be defined as “older” (Roberts et al., 2018), as it also was in the aforementioned publication (Biggs et al., 2022), we calculated the statistics for these comparisons and found a significant difference for all groups tested as presented in Table 3. We also present supplemental boxplots dichotomizing the patients at an age of 65 years. For the *m* = 3 and *m* = 5 settings with a 25 or 30 Hz low pass filter, the differences in PeEn were significant (*p* < 0.001) and the AUC indicated a relevant effect (AUC < 0.3). The plots and statistical information can be found in Supplementary Figures 1, 2 for study 1 and study 2.

**TABLE 3:** Area under the receiving operating characteristic values for the group comparison of old (>65 years) vs. young patients for different filter settings and embedding dimensions.

Data set 1 (*n* = 180)	Low pass	*m*	p (MWU)	AUC [95% CI]	PeEn old	PeEn young
n(old) = 76, n(young) = 104	25 Hz	3	<0.001	0.68 [0.60, 0.75]	2.06 (0.09)	2.02 (0.08)
	25 Hz	4	<0.001	0.68 [0.59, 0.75]	3.24 (0.20)	3.15 (0.18)
	25 Hz	5	<0.001	0.68 [0.60, 0.75]	4.54 (0.28)	4.36 (0.31)
	30 Hz	3	<0.001	0.70 [0.62, 0.77]	2.10 (0.10)	2.05 (0.09)
	30 Hz	4	<0.001	0.70 [0.62, 0.77]	3.33 (0.18)	3.21 (0.20)
	30 Hz	5	<0.001	0.70 [0.62, 0.78]	4.67 (0.29)	4.51 (0.34)
Data set 2 (*n* = 141)	25 Hz	3	<0.001	0.71 [0.62, 0.80]	1.61 (0.06)	1.57 (0.05)
n(old) = 55, n(young) = 86	25 Hz	4	<0.001	0.71 [0.62, 0.80]	2.23 (0.11)	2.17 (0.10)
	25 Hz	5	<0.001	0.72 [0.63, 0.81]	2.91 (0.16)	2.80 (0.15)
	30 Hz	3	<0.001	0.73 [0.63, 0.81]	1.64 (0.07)	1.60 (0.06)
	30 Hz	4	<0.001	0.73 [0.64, 0.81]	2.32 (0.13)	2.23 (0.11)
	30 Hz	5	<0.001	0.73 [0.64, 0.81]	3.04 (0.20)	2.91 (0.17)
Inhalational (*n* = 76)	25 Hz	3	0.001	0.72 [0.60, 0.84]	1.59 (0.07)	1.55 (0.05)
n(old) = 33, n(young) = 43	25 Hz	4	0.001	0.72 [0.60, 0.83]	2.87 (0.22)	2.74 (0.17)
	25 Hz	5	0.001	0.72 [0.60, 0.83]	2.87 (0.22)	2.74 (0.17)
	30 Hz	3	0.001	0.72 [0.60, 0.83]	1.62 (0.07)	1.58 (0.06)
	30 Hz	4	0.001	0.72 [0.60, 0.83]	2.98 (0.21)	2.85 (0.22)
	30 Hz	5	0.001	0.72 [0.60, 0.83]	2.98 (0.21)	2.85 (0.22)
Propofol (*n* = 65)	25 Hz	3	<0.001	0.82 [0.71, 0.92]	1.63 (0.04)	1.59 (0.03)
n(old) = 22, n(young) = 43	25 Hz	4	<0.001	0.85 [0.75, 0.93]	2.97 (0.13)	2.85 (0.12)
	25 Hz	5	<0.001	0.85 [0.75, 0.93]	2.97 (0.13)	2.85 (0.12)
	30 Hz	3	<0.001	0.85 [0.76, 0.94]	1.66 (0.04)	1.61 (0.05)
	30 Hz	4	<0.001	0.85 [0.75, 0.93]	3.11 (0.12)	2.95 (0.15)
	30 Hz	5	<0.001	0.85 [0.75, 0.93]	3.11 (0.12)	2.95 (0.15)

For each comparison the PeEn was significantly higher (indicated by AUC < 0.5 and the confidence interval excluding 0.5 as well as by *p* < 0.05), highlighting the consistent impact of age on PeEn. The PeEn values for the old and young are presented as median (interquartile range).

## Discussion

With our analyses we increase the knowledge regarding the effect of age-induced EEG changes on PeEn. It is well described that the EEG of an older patient under anesthesia shows lower amplitudes and faster oscillatory activity (Purdon et al., 2015; Kreuzer et al., 2020). This is likely the cause of the observed increase in the processed EEG indices of most monitoring systems (Ni et al., 2019; Obert et al., 2021, 2023). All these indices derive their information from the power spectrum of the EEG which is clearly affected by age (Schultz et al., 2004; Purdon et al., 2015; Kreuzer et al., 2020). The PeEn is an entropic time-domain measure that first made its way into anesthesia research because of its good performance to separate EEG signals recorded during wakefulness, from those recorded during anesthetic-induced unconsciousness (Jordan et al., 2008; Olofsen et al., 2008). The high performance seems to be based on the sensitivity of PeEn to evaluate changes in the faster frequencies. An in-depth investigation of PeEn with a *m* = 3 showed that the PeEn is correlated with the centroid of the power spectrum and also dependent on the peaks being observed in the raw signal (Berger et al., 2017). The counting of peaks was used almost 40 years ago in the parameter proposed by Kedem (1986). As stated in the introduction, contradictory results regarding the influence of age on PeEn have been published (Kreuzer et al., 2020; Biggs et al., 2022). As an explanation several factors were listed. With our results we could show that the embedding dimension and the choice of the low pass filter are not factors that could lead to PeEn being considered to be an age-independent parameter. We found that PeEn differed with age for the 30 Hz as well as the 25 Hz cut off, and for all the selected m values ranging from 3 to 5. Previous research into PeEn, dealing with the detection of different levels of anesthesia, showed that PeEn functions for different m and filter settings (Jordan et al., 2008; Olofsen et al., 2008; Liang et al., 2015). The difference in signal length is unlikely to be a factor, because as long as the signal remains stationary, the PeEn should not change for different lengths (Kreuzer et al., 2014). Furthermore, the longer the selected episodes, the slower the PeEn can react to sudden changes in the EEG. For instance in a case of intraoperative awareness even undetected episodes of more than 30 s may lead to an increased risk of memory formation (Dutton et al., 1995a,b). The longer the EEG segment used for PeEn calculation, the higher the risk of a delayed detection of sudden changes in the EEG. Especially during dynamic episodes like anesthesia induction and emergence, an EEG length of 30 s or more used for PeEn calculation could lead to a parameter lagging behind. Our findings were also independent of sample rate and type of maintenance anesthetic. Hence one main factor may be the anesthesia episode. We evaluated PeEn during unstimulated anesthesia maintenance. But an age-independent parameter should show this independent behavior for the entire anesthesia period. Another major difference was the sample size in the studies as well as the statistical approach. In the studies that were used here, more than 100 patients were included per study whereas there were 30 patients in the earlier study (Biggs et al., 2022). We evaluated the impact of age longitudinally by creating linear models, in contrast to the dichotomized approach with a cutoff of 65 years. Dichotomization of continuous data may come at a cost. The robustness of analysis and the conclusions drawn may suffer (Nafiu et al., 2015). Hence, we present the analysis of the continuous variable age using linear regression models as suggested in the literature (Altman and Royston, 2006). Further, with a small sample size, significant results will only be observed at large effect sizes. Hence we added the AUC to the dichotomized comparisons which we performed in order to reproduce the findings from Biggs et al. (2022). As can be clearly seen, the AUC decreased as more middle aged patients were included but because of our larger sample size, there was a significant difference even when the middle aged patients were included. The results were also largely independent of the maintenance anesthetic used as presented in Table 2. Hence, we cannot confirm the conclusion of PeEn being an age-independent parameter for anesthesia monitoring.

### Limitations

One difficulty in comparing these types of studies is that when very high induction rates are used the time course of the effect is very fast and does not generate stable conditions for analysis. In our opinion, propofol perfusions above 10 mg/kg/h lead to an overprediction of the Ce calculated by pharmacokinetic/pharmacodynamic models during the distributive phase (end front kinetics) resulting in very high Ce at LOC. Target controlled infusion does not correct the problem with inductions that include fast loading boluses, generating excessive cortical depression that will impact the results observed in EEG processing (Purdon et al., 2015; Kreuzer et al., 2020).

Our analyses were limited to one certain episode, but we are confident that the selected data is sufficient to convey our point of PeEn not being age independent. But as stated above, the PeEn should be calculated from rather short segments and then embedding dimensions of *m* > 5 could lead to an increased number of non-occurring patterns because the number of possible patterns is *m*! In our presentation of the linear models, we did not present “non-statistical” results in our slopes. There may be false negatives in this group as there may be false positives in the “significant” results. But in our case the experiments were conducted to present the general dependency of PeEn on age, which we could show with our data. Another issue that occurs when evaluating age induced changes is the dosing of the anesthetic. Older people require less anesthetic (Dundee et al., 1986; Shafer, 2000) this of course should be considered when evaluating the effects on the EEG. Therefore, studies used the age-adjusted MAC according to Mapleson (1996) for sevoflurane (Purdon et al., 2015; Kreuzer et al., 2020). For propofol, a linear decrease in propofol concentration was reported for older patients (Schultz et al., 2004; Purdon et al., 2015; Kreuzer et al., 2020). Another study just reports the decrease in drug concentration with age (Obert et al., 2023), since the MAC refers to a behavioral endpoint and may hence not reflect the hypnotic effects (Perouansky and Sleigh, 2021). That study did not show an age-related difference in PeEn (Biggs et al., 2022) when using target controlled infusion and the Schnider model (Schnider et al., 1999). While age-adjustment for steady state anesthesia may seem straightforward, the estimation of the effect site concentration during dynamic state transitions is another cup of tea. The Schnider model may overpredict during this stage and could also influence anesthesia maintenance (Coppens et al., 2010). Hence, at the state transition the real effect site concentration may not be correctly estimated and so age-adjustment may be not possible (Demaría, 2019).

## Conclusion

Based on our findings, we could show a strong dependency of permutation entropy (PeEn) on patient age, an effect that was independent of parameter, sample rate, and filter settings. Hence, age should be taken into consideration when using PeEn to monitor patient EEG.

## Data availability statement

The original contributions presented in this study are included in this article/Supplementary material, further inquiries can be directed to the corresponding author.

## Ethics statement

The studies involving human participants were reviewed and approved by the Local Ethics or Institutional Review Boards Waikato Hospital, Hamilton, New Zealand and the Klinikum rechts der Isar, Technical University of Munich, Germany. The patients/participants provided their written informed consent to participate in this study.

## Author contributions

DH and SK collected the data, analyzed the data, discussed the results, and wrote the manuscript. DO analyzed the data, discussed the results, and wrote the manuscript. GS and PS discussed the results and wrote the manuscript. JS and PG collected the data, discussed the results, and wrote the manuscript. MK designed the analysis, analyzed the data, discussed the results, and wrote the manuscript. All authors contributed to the article and approved the submitted version.

## References

[B1] Altman D. G. Royston P. (2006). The cost of dichotomising continuous variables. *BMJ* 332:1080. 10.1136/bmj.332.7549.1080 16675816 PMC1458573

[B2] Amrhein V. Greenland S. McShane B. (2019). Scientists rise up against statistical significance. *Nature* 567 305–307. 10.1038/d41586-019-00857-9 30894741

[B3] Bandt C. Pompe B. (2002). Permutation entropy: A natural complexity measure for time series. *Phys. Rev. Lett.* 88:174102. 10.1103/PhysRevLett.88.174102 12005759

[B4] Berger S. Schneider G. Kochs E. F. Jordan D. (2017). Permutation entropy: Too complex a measure for EEG time series? *Entropy* 19:692. 10.3390/e19120692

[B5] Biggs D. Boncompte G. Pedemonte J. C. Fuentes C. Cortinez L. I. (2022). The effect of age on electroencephalogram measures of anesthesia hypnosis: A comparison of BIS, Alpha Power, Lempel-Ziv complexity and permutation entropy during propofol induction. *Front. Aging Neurosci.* 14:910886. 10.3389/fnagi.2022.910886 36034131 PMC9404504

[B6] Coppens M. Van Limmen J. Schnider T. Wyler B. Bonte S. Dewaele F. (2010). Study of the time course of the clinical effect of propofol compared with the time course of the predicted effect-site concentration: Performance of three pharmacokinetic–dynamic models. *Br. J. Anaesth.* 104 452–458. 10.1093/bja/aeq028 20190259

[B7] Demaría M. (2019). Critical view of the effect site modelling of propofol. *Rev. Esp. Anestesiol. Reanim. Engl. Ed.* 66 425–433. 10.1016/j.redare.2019.03.012 31477336

[B8] Dundee J. Robinson F. P. McCollum J. Patterson C. (1986). Sensitivity to propofol in the elderly. *Anaesthesia* 41 482–485. 10.1111/j.1365-2044.1986.tb13271.x 3487990

[B9] Dutton R. C. Smith W. D. Smith N. T. (1995a). Brief wakeful response to command indicates wakefulness with suppression of memory formation during surgical anesthesia. *J. Clin. Monit. Comput.* 11 41–46. 10.1007/BF01627419 7745453

[B10] Dutton R. C. Smith W. D. Smith N. T. (1995b). Wakeful response to command indicates memory potential during emergence from general anesthesia. *J. Clin. Monit. Comput.* 11 35–40. 10.1007/BF01627418 7745452

[B11] Hentschke H. (2011). *Measures of Effect Size Toolbox*. *MATLAB.* Available online at: http://www.mathworks.com/matlabcentral/fileexchange/32398-measures-of-effect-size-toolbox (accessed June 4, 2023).

[B12] Hesse S. Kreuzer M. Hight D. Gaskell A. Devari P. Singh D. (2019). Association of electroencephalogram trajectories during emergence from anaesthesia with delirium in the post-anaesthesia care unit: An early sign of postoperative complications. *Br. J. Anaesth.* 122 622–634. 10.1016/j.bja.2018.09.016 30915984 PMC6465086

[B13] Jordan D. Stockmanns G. Kochs E. F. Pilge S. Schneider G. (2008). Electroencephalographic order pattern analysis for the separation of consciousness and unconsciousness: An analysis of approximate entropy, permutation entropy, recurrence rate, and phase coupling of order recurrence plots. *Anesthesiology* 109 1014–1022. 10.1097/ALN.0b013e31818d6c55 19034098

[B14] Kedem B. (1986). Spectral analysis and discrimination by zero-crossings. *Proc. IEEE* 74 1477–1493. 10.1109/PROC.1986.13663 10764936

[B15] Kreuzer M. Kochs E. F. Schneider G. Jordan D. (2014). Non-stationarity of EEG during wakefulness and anaesthesia: Advantages of EEG permutation entropy monitoring. *J. Clin. Monit. Comput.* 28 573–580. 10.1007/s10877-014-9553-y 24442330

[B16] Kreuzer M. Stern M. A. Hight D. Berger S. Schneider G. Sleigh J. W. (2020). Spectral and entropic features are altered by age in the electroencephalogram in patients under sevoflurane anesthesia. *Anesthesiology* 132 1003–1016. 10.1097/ALN.0000000000003182 32108685 PMC7159998

[B17] Liang Z. Wang Y. Sun X. Li D. Voss L. J. Sleigh J. W. (2015). EEG entropy measures in anesthesia. *Front. Comput. Neurosci.* 9:16. 10.3389/fncom.2015.00016 25741277 PMC4332344

[B18] Lutz R. Müller C. Dragovic S. Schneider F. Ribbe K. Anders M. (2022). The absence of dominant alpha-oscillatory EEG activity during emergence from delta-dominant anesthesia predicts neurocognitive impairment- results from a prospective observational trial. *J. Clin. Anesth.* 82:110949. 10.1016/j.jclinane.2022.110949 36049381

[B19] Mandrekar J. N. (2010). Receiver operating characteristic curve in diagnostic test assessment. *J. Thorac. Oncol.* 5 1315–1316. 10.1097/JTO.0b013e3181ec173d 20736804

[B20] Mapleson W. (1996). Effect of age on MAC in humans: A meta-analysis. *Br. J. Anaesth.* 76 179–185. 10.1093/bja/76.2.179 8777094

[B21] Nafiu O. O. Gillespie B. W. Tsodikov A. (2015). Continuous variable transformation in anesthesia: Useful clinical shorthand, but threat to research. *Anesthesiology* 123 504–506. 10.1097/ALN.0000000000000745 26114416 PMC4939840

[B22] Ni K. Cooter M. Gupta D. K. Thomas J. Hopkins T. J. Miller T. E. (2019). Paradox of age: Older patients receive higher age-adjusted minimum alveolar concentration fractions of volatile anaesthetics yet display higher bispectral index values. *Br. J. Anaesth.* 123 288–297. 10.1016/j.bja.2019.05.040 31279479 PMC7104362

[B23] Obert D. P. Schneider F. Schneider G. von Dincklage F. Sepulveda P. García P. S. (2023). Performance of the SEDLine monitor: Age dependency and time delay. *Anesth. Analg.* 1–9. 10.1213/ANE.0000000000006369 36727845

[B24] Obert D. P. Schweizer C. Zinn S. Kratzer S. Hight D. Sleigh J. (2021). The influence of age on EEG-based anaesthesia indices. *J. Clin. Anesth.* 73:110325. 10.1016/j.jclinane.2021.110325 33975095

[B25] Olofsen E. Sleigh J. W. Dahan A. (2008). Permutation entropy of the electroencephalogram: A measure of anaesthetic drug effect. *Br. J. Anaesth.* 101 810–821. 10.1093/bja/aen290 18852113

[B26] Perouansky M. Sleigh J. W. (2021). A crack at MAC. *Anesthesiology* 134 835–837. 10.1097/ALN.0000000000003761 33909884 PMC8119363

[B27] Purdon P. Pavone K. Akeju O. Smith A. Sampson A. Lee J. (2015). The ageing brain: Age-dependent changes in the electroencephalogram during propofol and sevoflurane general anaesthesia. *Br. J. Anaesth.* 115 i46–i57. 10.1093/bja/aev213 26174300 PMC4501918

[B28] Roberts A. W. Ogunwole S. U. Blakeslee L. Rabe M. A. (2018). *The population 65 years and older in the United States: 2016.* Washington, DC: US Department of Commerce, Economics and Statistics Administration.

[B29] Schneider G. Jordan D. Schwarz G. Bischoff P. Kalkman C. J. Kuppe H. (2014). Monitoring depth of anesthesia utilizing a combination of electroencephalographic and standard measures. *Anesthesiology* 120 819–828. 10.1097/ALN.0000000000000151 24694845

[B30] Schnider T. W. Minto C. F. Shafer S. L. Gambus P. L. Andresen C. Goodale D. B. (1999). The influence of age on propofol pharmacodynamics. *Anesthesiology* 90 1502–1516. 10.1097/00000542-199906000-00003 10360845

[B31] Schultz A. Grouven U. Zander I. Beger F. A. Siedenberg M. Schultz B. (2004). Age-related effects in the EEG during propofol anaesthesia. *Acta Anaesthesiol Scand* 48 27–34. 10.1111/j.1399-6576.2004.00258.x 14674970

[B32] Shafer S. L. (2000). The pharmacology of anesthetic drugs in elderly patients. *Anesthesiol. Clin. North Am.* 18 1–29. 10.1016/S0889-8537(05)70146-2 10934997

