# Supplementary material for: Permutation entropy is not an age-independent parameter for EEG-based anesthesia monitoring
Source: Front Aging Neurosci. 2023 Jun 15;15:1173304. doi: 10.3389/fnagi.2023.1173304 (PMC10308118; doi:10.3389/fnagi.2023.1173304)
Supplement: Supplementary file 1 [file Data_Sheet_1.docx]

Supplemental information for:

Permutation Entropy is not an age-independent parameter for EEG-based anesthesia monitoring


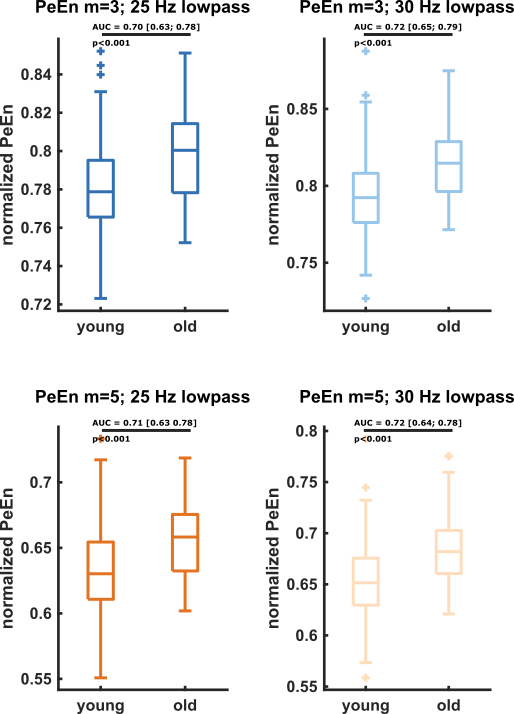


**Figure 1**: Boxplots comparing the young (<65 years of age) versus the old (>= 65 years of age) patients from study 1. For all settings the difference was significant (p<0.001) and relevant (AUC>0.70)


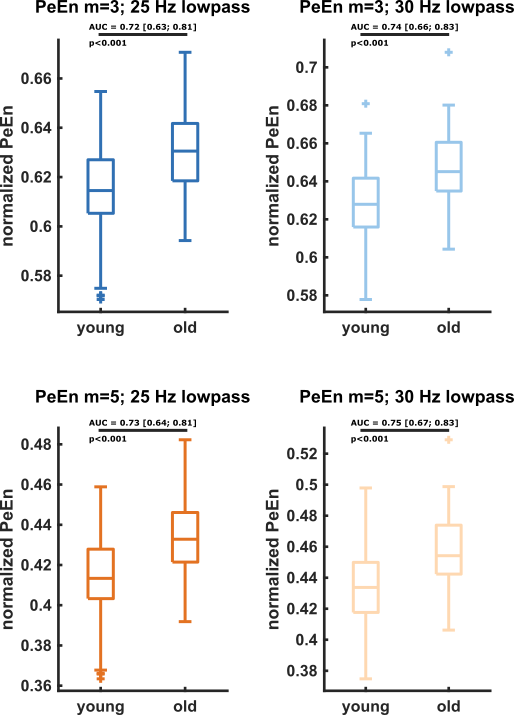


**Figure 2**: Boxplots comparing the young (<65 years of age) versus the old (>= 65 years of age) patients from study 2. For all settings the difference was significant (p<0.001) and relevant (AUC>0.70)
